# Cell Therapies for Acute Radiation Syndrome

**DOI:** 10.3390/ijms25136973

**Published:** 2024-06-26

**Authors:** Barbara A. Christy, Maryanne C. Herzig, Xiaowu Wu, Arezoo Mohammadipoor, Jennifer S. McDaniel, James A. Bynum

**Affiliations:** 1 Blood and Shock Resuscitation, US Army Institute of Surgical Research, Joint Base San Antonio, Fort Sam Houston, TX 78234, USA; maryanne.c.herzig.ctr@health.mil (M.C.H.); xiaowu.wu.civ@health.mil (X.W.); jennifer.s.mcdaniel7.civ@health.mil (J.S.M.); bynumj@uthscsa.edu (J.A.B.); 2 Department of Molecular Medicine, UT Health San Antonio, San Antonio, TX 78229, USA; 3 Hemorrhage and Vascular Dysfunction, US Army Institute of Surgical Research, Joint Base San Antonio, Fort Sam Houston, TX 78234, USA; arezoo.mohammadipoor.ctr@health.mil; 4 Department of Surgery, UT Health San Antonio, San Antonio, TX 78229, USA; 5 Trauma Research and Combat Casualty Care Collaborative, UT Health San Antonio, San Antonio, TX 78229, USA

**Keywords:** radiation injury, cell therapy, mesenchymal stromal cells, extracellular vesicles

## Abstract

The risks of severe ionizing radiation exposure are increasing due to the involvement of nuclear powers in combat operations, the increasing use of nuclear power, and the existence of terrorist threats. Exposure to a whole-body radiation dose above about 0.7 Gy results in H-ARS (hematopoietic acute radiation syndrome), which is characterized by damage to the hematopoietic system; higher doses result in further damage to the gastrointestinal and nervous systems. Only a few medical countermeasures for ARS are currently available and approved for use, although others are in development. Cell therapies (cells or products produced by cells) are complex therapeutics that show promise for the treatment of radiation injury and have been shown to reduce mortality and morbidity in animal models. Since clinical trials for ARS cannot be ethically conducted, animal testing is extremely important. Here, we describe cell therapies that have been tested in animal models. Both cells and cell products appear to promote survival and lessen tissue damage after whole-body irradiation, although the mechanisms are not clear. Because radiation exposure often occurs in conjunction with other traumatic injuries, animal models of combined injury involving radiation and future countermeasure testing for these complex medical problems are also discussed.

## 1. Introduction

### 1.1. Background

Countering the threat of a chemical, biological, radiological, nuclear, or explosives (CBRNE) attack is a global challenge [1,2,3,4,5]. Radiological or nuclear threat is best exemplified by the consequences of the bombing of Hiroshima and Nagasaki and by the nuclear reactor disasters at Chernobyl and Fukushima and the mass casualties that resulted [6]. Other radiological threats exist in the form of dirty bombs, improvised explosive devices intended to disseminate radiological hazards [7,8,9,10,11]. The Radiation Injury Treatment Network (RITN) was begun in 2006 to provide a resource to help following a mass casualty incident (ritn.net). The U.S. military has a particular interest in medical countermeasures (MCMs) for radiation injury due to the increased exposure risk to military personnel, either in combat operations or in mass casualty events where active duty or National Guard troops may be called upon to provide assistance [12,13]. Currently, there are a small number of FDA-approved MCMs for the treatment of mild-to-moderate radiation-induced injuries, but the development of additional effective countermeasures or prophylactics to mitigate these increased risks is an urgent need.

Radiation injury frequently occurs in conjunction with other types of injury, such as burns and other types of trauma. This is especially true in terrorist attacks, industrial accidents, or combat involving nuclear weapons. There is currently a gap in knowledge regarding treatment for patients who have sustained a combined injury involving radiation along with other types of trauma simultaneously. One challenge for investigating combined injury in animal models is that it is difficult to mimic multiple injuries at the same time (such as those caused by blast). The experimental sequence and timing of injuries likely affect the outcome [14]. Regardless, it is important to investigate the effects of radiation exposure on the physiological response to different types of trauma including burn wounds, and, conversely, it is also necessary to investigate the effects of additional trauma on morbidity and mortality in patients with radiation injury. Future studies should inform clinical practice in radiation-combined injury patients by evaluating the impact on healing processes and timelines and by re-examining current guidelines for triage and treatment of traumatic injuries with the addition of radiation exposure.

### 1.2. Preclinical Models

Although the need for the development and approval of treatments for acute radiation exposure is urgent, this is a difficult and lengthy task. In developing treatments or prophylactics for FDA approval, reliable reproducible animal models are essential. Due to ethical and humanitarian concerns, no clinical trials are possible for acute radiation syndrome, meaning that the “animal efficacy rule” of the FDA applies here [15,16]. The “animal efficacy rule” states that “FDA’s regulations concerning the approval of new drugs when human efficacy studies are not ethical and field trials are not feasible are codified in 21 CFR 314.600 through 314.650 for drugs and 21 CFR 601.90 through 601.95 for biological products”. In the application of this rule, the FDA requires a well-understood pathophysiological mechanism, as well as a demonstration of the effects in at least two well-characterized animal models. No animal model completely mimics human anatomy and physiology; therefore, testing in more than one species is required. Although clinical trials are not needed, this high standard means that the path to approval is still lengthy and expensive. There are currently several well-characterized animal models in multiple species (Table 1; [17,18,19]). While none of these models can completely recreate human physiology, each has its own set of advantages and disadvantages.

Nonhuman primates (NHP) are the most obvious choice to most closely mimic human physiology, but they must be used judiciously due to ethical and cost considerations [20]. For practical reasons, small animal models are usually used to test medical countermeasures (MCM) initially, before moving on to testing in larger animal models with a much smaller subset of treatments [17,18].

Because of their small size, fast generation times, and low cost for maintenance, rodent models (mouse or rat) are commonly used. Most academic institutions are well-equipped to house and care for rodents. As a less sentient species, approval for use is more easily justified and obtained. Lastly, because of their lower cost to purchase and maintain, larger numbers can easily be utilized leading to more accuracy and consistency. The larger size of rats makes them a good model for trauma studies testing surgical interventions or for studies requiring more tissue or a greater volume of blood. On the other hand, mice are the most commonly utilized species and, as such, are better characterized, have more commercially available antibody reagents, and can be more easily manipulated genetically in order to determine the importance of particular molecular pathways for the radiation response.

### 1.3. Combined Injury Animal Models

There are multiple animal models available to begin to study the management of combined injury involving radiation exposure. Palmer et al. described a mouse model combining sublethal whole-body irradiation and significant (15% TBSA) scald burn wounds [21]. In this model, higher mortality was observed with the combined injury compared with radiation alone, as well as a greater decrease in circulating white blood cells at 48 h post injury. Higher circulating levels of the proinflammatory cytokines, interleukin-6, and tumor necrosis factor-a were also observed. Medhora et al. described a rat model for combined radiation and skin wound injuries that can be used to evaluate the effects of wound trauma on both acute and delayed radiation effects [22]. This model was built on a method used in a previous study [23] to evaluate combined whole thorax lung irradiation and nonlethal soft X-rays to the skin and measured the incidence of radiation pneumonitis (lung injury). Results from the study indicate that skin irradiation 3 h after thorax irradiation decreased radiation pneumonitis. A more recent study [22] used partial-body shielding of one leg to spare a minimal amount of bone marrow (~8%) from the highest dose of radiation, which allowed at least some of the animals to survive the acute hematopoietic syndrome so that later effects could be studied. Full-thickness skin wounds were generated by punch biopsy to mimic puncture wounds that might occur in a radiological incident. It was noted that the addition of skin wounds increased mortality in the animals with combined injury. Skin wound healing was also delayed in animals with combined injury compared with animals that received only skin wounds, but delayed radiation effects (pneumonitis and nephropathy) were not altered by the presence of skin wounds. Treatment with the drug lisinopril (started at 7 days post injury) did not affect wound healing or early mortality but did decrease morbidity due to delayed radiation effects. Additional mouse combined injury models have been described, including a model combining radiation injury with hemorrhage [24] and another combining radiation injury and a skin wound [24,25]. In these models, additional injuries in addition to radiation caused increased mortality and/or morbidity. When 15% TBSA skin wounds were combined with whole-body radiation exposure, significantly higher mortality occurred compared with radiation injury alone over a 30-day observation period [26,27]. Both radiation injury alone and the combined injury resulted in bone marrow cell depletion, weight loss, and pancytopenia. These and other combined injury models will be important for the determination of the interactions between radiation exposure and other types of trauma occurring in the same patient and will inform clinical practices for the treatment of these complicated situations.

It is surprising to note that skin injury prior to radiation exposure in mice decreases mortality rather than increases it, at least under some conditions [14,23,28,29]. This protection even extended to subcutaneous wounding shortly after X-irradiation and was termed “protective wounding” [30,31]. The survival benefit delivered by even a small subcutaneous cut (3 mm) was attributed to enhanced and accelerated recovery of hematopoiesis and increased production of some cytokines [31]. Using these and other yet-to-be-described models (for example, radiation plus cold injury or radiation plus blast injury), the interactions of various types of trauma and radiation injury can be studied, as well as the efficacy of cellular therapies and other potential radiomitigators in these settings. It is also clear that the complexities of the combined injury models will make the interpretation and development of countermeasures more difficult.

## 2. Acute Radiation Syndrome

**Figure 1:**
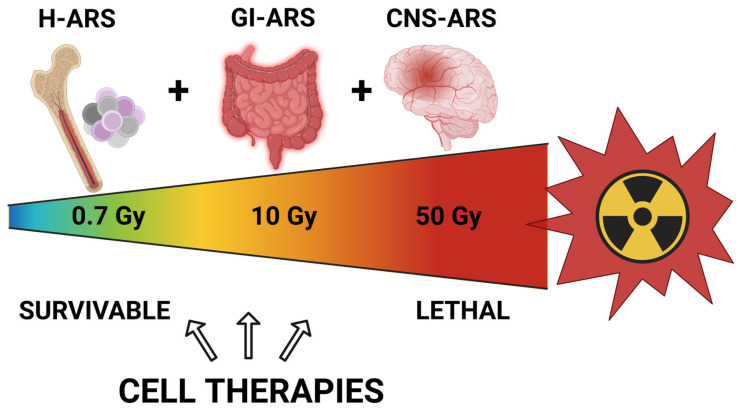
Subsyndromes of acute radiation syndrome. Whole-body radiation doses above approximately 0.7 Gy (in humans) result in serious radiation injury, with the hematopoietic system being affected at the lower dose range. Above 10 Gy, damage to the gastrointestinal system is observed in addition to the hematopoietic system damage. Above 50 Gy, serious damage to the central nervous system also occurs. As indicated in the figure, cell therapies may provide benefits to patients with H-ARS and/or GI-ARS but are unlikely to benefit patients with CNS-ARS. ARS, acute radiation syndrome. H-ARS, hematopoietic ARS. GI-ARS, gastrointestinal ARS. CNS-ARS, central nervous system ARS.

Exposure to high doses of penetrating ionizing radiation, whether gamma rays, neutrons, or high-energy X-rays, is extremely damaging to biological systems. In a nuclear blast or following the use of a nuclear weapon, all or most of the body can be irradiated with a high dose in a relatively short period of time. If a whole-body radiation dose of more than approximately 0.7 Gy is delivered, this causes a severe acute illness called Acute Radiation Syndrome (ARS, also called radiation toxicity or radiation sickness). Due to natural repair processes, it is less toxic if the same overall dose is accumulated in smaller doses over a longer period of time. As illustrated in Figure 1, there are three defined but overlapping ARS subsyndromes that occur based on the dose received [32,33,34]. It should be noted that there is a gradient of damage seen with increasing doses. Although the injury response will vary due to individual patient differences (age, body size and composition, gender), even exposure to relatively low doses will likely produce harm. Patients exposed to at least 0.7 Gy develop symptoms reflective of bone marrow syndrome or hematopoietic syndrome (H-ARS). In this syndrome, the bone marrow and blood cells are primarily impacted. There is a drop in the numbers of all types of blood cells—pancytopenia—and the primary causes of death within this group are infection, coagulopathy, and hemorrhage. Some of the affected individuals will be expected to survive with supportive therapy allowing repopulation of the blood and bone marrow, especially at the lower end of the dose range, but recovery can take weeks to years. In fact, long-term residual bone marrow damage has been described in mouse model survivors of H-ARS with decreased white blood cells, lymphocytes, red blood cells, and platelets compared with nonirradiated controls even 20 months post irradiation [35,36,37,38]. Patients exposed to more than 10 Gy develop gastrointestinal syndrome (GI-ARS) in addition to blood and bone marrow damage. High doses of radiation cause endothelial and epithelial cell dysfunction and increased vascular permeability, which is especially damaging in the GI system [39]. Symptoms observed in GI-ARS include vomiting, diarrhea, electrolyte imbalance, and dehydration, and this syndrome is often lethal, especially at higher exposure levels. Supportive care may increase survival at the lower end of the dose range. A cardiovascular/central nervous system syndrome (CNS-ARS) will develop in addition to the damage to the hematopoietic and GI systems when patients are exposed to extremely high levels of whole-body radiation of more than 50 Gy. Individuals with this level of injury die within a few days, and no treatment options are currently available. A fourth syndrome has also been described, Cutaneous Radiation Syndrome (CRS) (https://www.cdc.gov/nceh/radiation/emergencies/pdf/ars.pdf; accessed on 24 May 2024) [40]. In CRS, exposure is limited to a smaller area of the body and causes mainly skin damage. Patients with cutaneous skin damage alone do not exhibit the severe life-threatening symptoms seen in ARS, but large skin doses can cause permanent damage to the affected area. Triage for radiation exposure includes severity scoring for prioritization for the use of medical countermeasures [41].

## 3. Management of Acute Radiation Syndrome

### 3.1. Background

Currently, there are a limited number of treatments beyond supportive therapies for the treatment of ARS [33,42]. The development of additional MCMs for the treatment of ARS is a priority [15,38,43,44,45,46,47,48,49,50,51]. MCMs for ARS fall into one of three categories: radioprotectors, radiomitigators, and radiotherapeutics [52]. Radioprotectors are treatments that are administered before radiation exposure and offer some protection against harmful effects. These interventions may be beneficial when the risk of significant exposure is likely, such as for medical and safety personnel responding to a nuclear incident, or for military personnel in combat situations where advanced weaponry is likely to be utilized. Radioprotectants are less useful when exposure is not anticipated prior to the incident, such as in a terrorist attack or nuclear facility incident. Radiomitigators are used after radiation exposure but prior to the development of ARS symptoms. To be classified as a radiomitigator, the treatment should be able to prevent or reduce eventual tissue and organ damage caused by exposure. Although administered after the initial insult has occurred, a radiomitigator might interrupt the cycle of immune, inflammatory, or other host responses that can lead to further damage. Alternatively, radiomitigators could be agents that act by initiating or accelerating regenerative processes to enable victims to survive the initial coagulopathy, immune dysfunction, and loss of intestinal barrier function. If a treatment of this type could be stockpiled and utilized rapidly in mass casualty situations, morbidity and mortality might be significantly reduced. Radiotherapeutics are administered once ARS symptoms have appeared to treat the symptoms. Maximum benefit to reduce mortality will likely be achieved by early treatment to prevent or reduce some of the damage before severe symptoms occur.

### 3.2. Approved Therapies

No agents have been fully approved by the FDA for use as radioprotectants at this time, but several recombinant growth factor agents have been approved for emergency use as radiomitigators, including Neupogen^®^ Neulasta^®^, Nplate^®^ (all from Amgen, Thousand Oaks, CA, USA), Leukine^®^ (Partner Therapeutics, Lexington, MA, USA), Udenyca^®^ (Coherus Biosciences, Redwood City, CA, USA), Stimufend^®^ (Fresenius Kabi, Bad Homburg, Germany), and Ziextenzo^®^ (Sandoz, Basil, Switzerland) (https://www.accessdata.fda.gov/emergency-preparedness-and-response/mcm-issues/radiological-and-nuclear-emergency-preparedness-information-fda#mcms, accessed on 5 May 2024). These are approved for H-ARS specifically and work by facilitating recovery of bone marrow precursors to generate neutrophils and white blood cells involved in immune function. They have a well-established history of clinical use for other indications and well-characterized safety profiles or are biosimilar to drugs with those features. There are limitations and side effects with all these agents: they do not work as radioprotectants; they may require additional supportive care which may not always be available; and they may cause delayed ARDS (Acute Respiratory Distress Syndrome). Another promising treatment that has not yet been approved, Entolimod (CBLB502), is currently under development. Entolimod (CBLB502) is a shortened version of the protein flagellin, which acts as a stimulatory ligand for host toll-like receptor (TLR) receptor signaling without the immunogenicity and toxicity of full-length flagellin [53]. TLR signaling stimulates immune function and induces NF-κB signaling, which in turn is antiapoptotic. CBLB502 has shown promise as a radiation countermeasure in murine and nonhuman primate (NHP) models [15]. Interestingly, the drug was shown to exert both radioprotective and radiomitigative effects in murine testing [54,55,56], making it even more attractive as a radiation MCM.

## 4. Cellular Therapeutics

### 4.1. Background

Cellular therapy describes the therapeutic use of not only live cells but also the use of cell products [57,58,59]. Transfusion with whole blood or blood products and hematopoietic stem cell transplantation (HSCT) can be classified as cellular therapies and these methods are already used in patients with radiation injury [42,60,61,62]. When the hematopoietic system is compromised in H-ARS, one of the earliest indicators of serious radiation damage is pancytopenia [34,63]. Bone marrow damage to precursor and stem cells blocks the replenishment of necessary blood cells, including white blood cells, red blood cells, and platelets, resulting in susceptibility to infection, clotting dysfunction, and diminished ability to recover from the damage. Blood transfusion is a temporary way to replenish some of the important cell types to buy time and allow the hematopoietic system to regenerate, assuming any stem and precursor cells remain in the bone marrow. A more comprehensive radiation treatment is bone marrow transplantation or hematopoietic stem cell transplantation [61,64,65,66]. Bone marrow contains hematopoietic stem cells (HSCs) involved in replenishing all the cell types in the blood. HSCs can also be found circulating in peripheral blood or umbilical cord blood. Umbilical cord blood may be a practical source for isolation based on availability due to its classification as medical waste. For radiation injury, HSCs need to come from an unirradiated donor source (allogeneic). Because of their immunogenicity, HSCs should be HLA-matched to reduce the chance of developing life-threatening graft v. host disease (GvHD) [67].

There is an intense interest in the use of cellular therapies beyond conventional blood transfusion for the mitigation or treatment of radiation injury [59]. Radiation injury can be complex, involving multiple organ systems, and as such may require a complex therapy consisting of multiple agents to address the damage [58]. The administration of either live cells or cell-produced biological products such as the secretome or extracellular vesicles (EVs) may provide the desired broad benefits (described below). Beyond the standard treatments of blood transfusion and hematopoietic stem cell transplantation, cellular therapies derived from mesenchymal stromal cells (MSCs) are at the forefront, especially for H-ARS.

#### Mesenchymal Stromal Cells—Background

Although they are not the only progenitor cell type under consideration for cell therapies, human MSCs have historically been the most studied cells for clinical use. Over 1000 human clinical trials have been conducted or are currently being conducted using MSCs for a variety of different diseases and injuries [68,69,70,71]. MSCs are adult stem-like cells and avoid the ethical considerations involved in the use of embryonic or neonatal cells. MSCs can be easily derived and expanded from readily available tissue sources such as bone marrow, adipose tissue, placental tissue, umbilical cord, or cord blood. Individual MSC populations show some differences reflective of their tissue source [72,73,74,75] and individual donor characteristics [76,77], but they share many similarities. MSCs are primary cells and are not immortalized cell “lines”; therefore, they have a limited lifespan and a reduced oncogenic risk. Although there is no one marker characteristic of MSCs, cells must conform to certain minimal criteria defined by a working group of the International Society for Cell and Gene Therapy (ISCT) to be classified as MSCs [78]. These criteria include expression of relevant surface proteins (≥95% positive for CD90, CD105, CD73) and a lack of expression of surface proteins characteristic of other cell types (≤2% positive for CD11b, CD34, CD14, CD45, CD31). The cells should also express undetectable levels of MHC Class II antigen on their surface under normal culture conditions; this property makes the cells less immunogenic (immune-privileged) and is important for safety reasons. However, under inflammatory conditions, likely similar to those found in patients with radiation injury, MHC Class II surface antigen expression increases in MSCs [79,80]. To be classified as MSCs, the cells must also be able to attach and proliferate on tissue culture plastic under standard conditions and retain the ability to differentiate into multiple lineages such as osteocytes, adipocytes, and chondrocytes under permissive conditions.

MSCs have anti-inflammatory properties and immunomodulatory activity and may also have antifibrotic and pro-regenerative properties, suggesting they may be beneficial in mitigation and/or treatment of ARS. MSCs and other cellular therapeutics secrete cytokines and other factors, proteins, RNA, and lipids, many of which are contained within EVs. These secreted components act in a paracrine manner to attenuate inflammation, augment angiogenesis, stimulate tissue repair, and modulate immune function (see [71] for an excellent review). Although not completely reflective of in vivo activity, a number of in vitro assays designed to assess the potency of different MSC preparations have been developed and are in common use [81,82,83,84,85]. Clinical trials have been performed for many different patient indications, including cardiovascular disease, immune disorders, neurological disease, osteoarthritis, and even as a treatment for COVID-19 [69]. MSCs have been approved for use in two inflammatory diseases, acute GvHD and Crohn’s disease [85,86]. Because they are relatively immune-privileged and immune-evasive, allogeneic MSCs can be used “off the shelf” in unrelated nonmatched donors, a property that dramatically increases their usefulness and cost-effectiveness. MSCs have shown an excellent safety profile, even when administered intravenously to patients [87,88,89,90,91]. Although there were concerns regarding potential procoagulant activity when MSCs or EVs are infused directly into the bloodstream of nonmatched recipients [92,93,94,95,96], these concerns have mostly been allayed by the addition of anticoagulants to the cells at the time of infusion [97,98].

Many different factors including immunomodulators have been identified as MSC-secreted factors, but it is not entirely clear what factors or combination of factors is responsible for activity in modulating immune function and inflammation [99,100,101,102,103]. The use of live MSCs rather than a mix of defined factors is more complicated but allows a more sophisticated response and the ability to adapt to different needs and conditions found in individual patients. Although MSCs secrete many factors responsible for much of their activity, they can also interact directly with immune cells in vitro [83,104,105,106,107]; interaction with host immune cells following infusion into animals likely provides additional functional activity. On the other hand, the use of live cells limits the applicability of the treatment method to well-equipped hospitals and clinics that have the necessary expertise and equipment to prepare and administer the cells under sterile conditions [70,80,108,109,110]. For this reason, investigation into the use of cell-free products derived from the cells is very important. Cell-free products produced by cells include secretome or conditioned medium and more purified EVs derived from conditioned medium [99,101,111,112,113,114,115]. It is possible that cell-free products such as secretome/MSC-conditioned medium or EVs could be packaged in a form (lyophilized or freeze-dried) that would be stable and readily available to stockpile for use in a future mass-casualty event or combat situation [114,116,117]. For that reason, investigation into the efficacy and optimal packaging and administration route for these products will be very important [118,119,120]. However, the choice of cell type and conditions for the cells producing the cell-free products will still influence the cell-free product and therefore remains an important issue.

### 4.2. Cellular Therapies for Radiation in Animal Models

#### 4.2.1. Cells

Multiple published studies in rodent models have shown the benefit of cellular therapies for the treatment of acute radiation injury (Table 2). In mouse models, several studies have demonstrated a survival benefit with live mouse MSCs using IV or IP administration following whole-body irradiation (gamma or X-ray) with a radiation dose that would normally be lethal [81,121,122,123]. Lange et al. showed 30–88% survival at 7 months vs 100% mortality of untreated animals irradiated with 9.5 Gy when treated 8 h after irradiation with 10^6^ mouse bone-marrow-derived MSCs (BM-MSCs) [121]. Yang et al. found that while 7 Gy irradiation gave 20% survival at 45 days, infusion with 10^6^ mouse MSCs resulted in improved 45-day survival of 50–60% [122]. Francois et al. determined that the MSCs need not be MHC-matched to be tolerated and mitigate radiation damage; MSCs derived from either syngeneic or allogeneic mice both showed positive effects [123]. The study by Chinnadurai et al. showed that “licensed” MSCs (activated by pretreatment with interferon gamma) were retained better than nonlicensed MSCs, although the difference was only seen in nonirradiated animals. The “licensed” MSCs protected irradiated mice from ARS, with 72% survival at day 30 versus 30% survival for vehicle-treated mice. In this study, male mice were noted to be more radiosensitive than female mice, and radiation treatment was performed at two doses of 4 Gy each. Two large doses of 10^7^ MSCs were given at 24 h and 8 days after irradiation; IP injection was utilized to allow tolerance of the larger dose [81]. IP injection of mouse-adipose-derived stem cells also increased survival in lethally irradiated mice [124]. In this study, two different strains of mice were used to compare allogeneic (cross-strain) and syngeneic (same strain) MSCs. Thirty-day survival after 9.25 Gy irradiation increased with an IP injection of 5 × 10^6^ cells 24 h post irradiation. Beyond MSCs, other cell types have also shown benefits in lethally irradiated mice. Mouse endothelial cells (ECs) or their EV products (EC-EVs) increased both survival and bone marrow cellularity in irradiated mice [125].

Cells from human sources have also been tested in mouse radiation injury models. Increased survival of lethally irradiated mice was observed after retro-orbital administration of human BM-MSCs [64], umbilical-cord-blood-derived MSCs (UCB-MNCs) [127], or IV administration of human UCB-MSCs [128,131]. The study by Diaz et al. utilized a mouse total body irradiation model developed by Orschell’s group [36,137,138]. Mice were irradiated at 8 Gy for a lethal dose of 30% by 30 days. Human BM-MSCs (1.2 × 10^7^ per kg body weight) were administered by retro-orbital injection at either 3 h or 30 h post irradiation and monitored for 30-day survival, body weight change, and pancytopenia. Survival increased, although there was no change in body weight or pancytopenia, suggesting that recovery of the hematopoietic system was not the reason for increased survival. Similarly, the retro-orbital injection of human umbilical cord blood mononuclear cells (UCB-MNCs) increased 50-day survival in mice from 20% to 92% when given with antibiotics [127]. Antibiotics were given 4 h after irradiation with a lethal dose of 9 Gy; 2 × 10^8^ cells were administered retro-orbitally in four doses given between 24 and 52 h post irradiation. These benefits were not limited to the retro-orbital delivery of human cells. Kim et al. performed IV administration of human UCB-MSCs into mice at 1 × 10^6^ cells/dose at 3 and 30 h post 6 Gy irradiation and also achieved improved survival (from 0% in untreated animals at day 26 to approximately 40% survival in MSC-treated animals) [131]. Human macrophages or monocytes also act as radiomitigators in lethally irradiated mouse models. Interestingly, human macrophages that have been “educated” (preconditioned) by co-culturing with human MSCs are more potent at reducing radiation effects in a mouse model than either MSCs alone or “uneducated” macrophages [130]. It was subsequently shown that incubation of the macrophages [132] or even undifferentiated monocytes [133] with EVs derived from human MSCs could substitute for the MSC co-culture step. When the EVs used for “educating” the macrophages or monocytes were derived from MSCs that had been activated by treatment with the TLR4 receptor agonist lipopolysaccharide (LPS, a presumed inflammatory signal), the resulting “educated” macrophages were much more potent at increasing/prolonging survival, increasing expression of several key factors and reducing radiation damage to the spleen and bone marrow [132,133]. Administration of these cells was effective when given at either 4 h or 24 h post irradiation but was not effective at 48 h [133].

Although increased survival has been observed in these studies following the administration of MSCs or other cell types, there is debate about the mechanism. Lange [121] demonstrated that intravenous administration of mouse bone marrow MSCs within 8 h of irradiation promoted both overall survival and hematopoietic recovery in lethally irradiated mice; similar rescue of hematopoiesis was observed by Kim et al. [131] using human UCB-MSCs administered by IV infusion at either 3 h or 30 h post irradiation. In contrast, the recent study by Diaz et al. [64] demonstrated increased survival of lethally irradiated mice following retro-orbital injection of human BM-MSCs but observed no difference in pancytopenia or bone marrow hypocellularity in the treated animals. In that study, MSCs appeared to protect the gut from damage after irradiation. Although the studies use different models, injury conditions, cell types, doses, methods, and timing of cell administration, the consensus indicates that MSCs or other progenitor cells can confer survival benefits in rodents receiving potentially lethal doses of radiation. It also appears that cells from either the same species (either syngeneic or allogeneic) or from humans (xenogenic) are safe and show benefit. It is not yet clear what the optimal method of administration is. Although many studies utilize intravenous infusion, larger cell doses appear to be tolerated when administered intraperitoneally [81]. In the studies described here, cells administered by IV infusion, IP injection, or retro-orbital injection all decreased mortality due to radiation damage. The addition of prophylactic antibiotics has been suggested to confer additional survival benefit [123,127], likely by decreasing mortality due to infection during recovery of the hematopoietic system.

Interestingly, previous work has suggested that the inclusion of human MSCs along with HSCs in transplantation may facilitate engraftment and accelerate hematopoietic recovery [139,140]. In mice exposed to a lethal dose of radiation, the addition of mouse MSCs along with bone marrow cells (including HSCs) increased survival and promoted hematopoietic recovery [141]. However, the benefits were only seen up to a point; the addition of a large dose of MSCs was detrimental. More evaluation of these types of combination therapies is clearly warranted.

#### 4.2.2. Cell Products

Conditioned medium collected from cultured cells, also called the secretome, contains many biologically active components, including EVs [99,101,113]. EVs are small membrane-enclosed vesicles secreted from cells; they can be found naturally in blood and other biological fluids or in the secretome of cultured cells. The contents of EVs include many biologically active molecules, including proteins, peptides, hormones, lipids, RNA, and other signaling molecules [101,103,113,115,142]. The composition of secreted EVs likely differs in different cell types and under different cellular conditions [101,108,111]. It is thought that much of the activity ascribed to MSCs is mediated through a paracrine mechanism via EV secretion. EVs can be further purified and concentrated from the secretome, and biological fluids and have been tested in multiple animal models of disease and have shown to be effective for the treatment of several conditions [143,144]. EVs are also being tested in a number of clinical trials [144]. Conditioned medium from rat MSC cultures has been tested in rat models of partial radiation injury. In one study, rats were administered conditioned medium from rat MSCs after abdominal irradiation (IP administration) in order to study intestinal damage. Rats receiving conditioned medium showed improved intestinal damage and survival, and medium from “activated” MSCs proved to be more potent [135]. Another study showed that conditioned medium from rat MSCs improved liver pathology when given prior to liver irradiation in rats [136]. In preclinical studies of radiation damage, EVs derived from “primed” MSCs showed protective activity [132,133]. In these studies, EVs derived from human MSCs can alter monocytes/macrophages to produce hematopoietic recovery benefits in a lethal mouse irradiation model for H-ARS. Additional evidence indicates that EVs derived from mouse bone marrow endothelial cells [125], human dental pulp stem cells [134], or human MSCs [129] increase survival, reduce bone marrow damage, and promote hematopoietic recovery in irradiated mice. Interestingly, Schoefinius et al. demonstrated that both mouse BM-MSCs and the EVs secreted by them conferred long-term survival benefits to lethally irradiated mice [126]. However, the MSCs were able to confer additional benefits in the short term. Their conclusion was that EVs protected irradiated hematopoietic stem cells but not progenitors. Finally, in a rat model partial-body knee irradiation, both rat BM-MSCs or their secreted EVs showed efficacy for reducing radiation-induced bone loss and bone marrow adiposity [145]. Although it is difficult to compare the efficacy of cells and their EV products because they have not often been compared in the same experiment, the reported benefits seen using EVs are encouraging and should be pursued further. Effective EV treatments would be much more practical for use on the battlefield or in military field hospitals, because of the potential for lyophilization for easier transport and storage. However, it remains to be seen whether EV function can be maintained in the lyophilized form. Another caveat is that EVs are not live cells; they cannot respond to environmental conditions. It will therefore be important to determine how to best induce production of the most clinically active EV products. For example, MSCs may secrete more potent anti-inflammatory EVs if they are activated by pretreatment with inflammatory mediators such as interferon-gamma or LPS [133,146].

**Figure 2:**
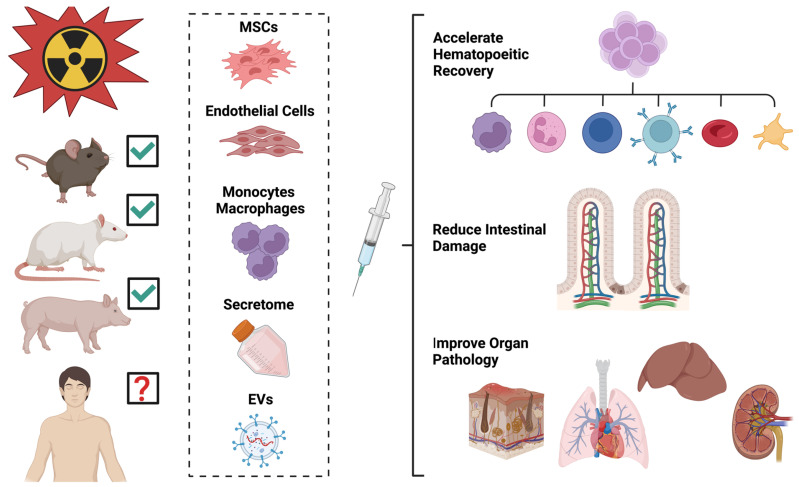
Cellular therapeutics (cells or cell products) have shown benefits in several organ systems in animal models of acute radiation syndrome.

The bulk of cellular therapeutic studies for acute radiation syndrome has been conducted in small animal models. Systemic administration of cells or EVs is more costly in larger animals due to the much larger body size and blood volume. However, porcine models are useful for the investigation of cellular therapeutics in the treatment of combined injuries that involve radiation and skin wounding. A minipig model was used to evaluate the efficacy of autologous adipose-derived regenerative cells (ADRCs, a heterogenous population including MSCs) on wound closure after sublethal whole-body irradiation combined with a full-thickness burn injury. Wound closure was accelerated in animals treated with ADRCs administered either by local injection or intravenously [147]. It is likely that cellular therapeutic treatments showing benefit in rodent models will continue to be tested and validated in large animals, including both porcine and nonhuman primate models. Thus far, cellular therapeutics have shown benefits in multiple animal models, but it remains to be seen if these agents will benefit human patients with radiation injury (Figure 2).

#### 4.2.3. Countermeasure Testing in Combined Injury Models

Several countermeasures have been tested in combined injury models. In the mouse radiation + burn model described by Kiang et al., ciprofloxacin (given daily for 21 days) was successful in increasing survival [27]. Treatment significantly reduced early (Day 1) γ-H2AX focus formation indicative of DNA damage and later proinflammatory cytokine expression (Day 10). In another study, treatment of mice subjected to combined radiation + skin wound injury with the thrombopoietin receptor agonist Alxn4100TPO improved survival and mitigated weight loss, although wound healing was delayed [148,149]. Treatment with the hunger-stimulating peptide hormone ghrelin improved survival, mitigated weight loss, and accelerated wound healing in the mouse combined radiation + skin wound model [150]. Ghrelin treatment was also effective in mice subjected to radiation + 15% TBSA burn injury, increasing survival and mitigating pancytopenia and bone marrow cell depletion [150]. Cellular therapy using mouse bone marrow MSCs has also been tested using this model [26]. In this study, IV administration of MSCs grown under hypoxic conditions 24 h after combined injury led to a 30% increased 30-day survival, attenuated weight loss, accelerated wound healing, and decreased bone marrow cell loss. In contrast to other studies (Table 2), this group did not find the benefit of MSC treatment in mice treated with radiation alone in their model, but MSCs did appear to provide benefits to animals with combined injury [26]. Kiang et al. [151] also observed another difference between radiation injury alone and combined radiation + burn injury. The approved radiomitigator pegylated G-CSF improved survival, reduced weight loss, mitigated WBC and platelet depletion, and blocked splenomegaly in mice subjected to radiation injury alone but did not benefit mice with combined injury. This suggests that treatments that work for radiation injury alone may or may not work for combined injuries, and that treatments providing benefit in combined injury models may not provide benefit in animals with radiation injury alone. Determination of the best treatments for radiation alone and for different types of combined injury will require more careful study.

#### 4.2.4. Delivery of Cellular Therapeutics

Cells or their products can be introduced by several means, including intravenously, intrathecally, topically, intraperitoneally, subcutaneously, intramuscularly, or by direct cardiac implantation [152]. It is not yet clear which route of administration is superior, and the choice of administration route may depend at least in part on patient indications [70,152]. For example, because IV-administered cells quickly become sequestered mainly in the lung, this may be the preferred route of administration for acute lung injury therapies. Conversely, topical application for burns or other skin wounds may be more suitable. At this time, the preferred administration route for ARS is unclear, and this topic will need further exploration. The most common routes of administration in preclinical animal testing for ARS caused by whole-body irradiation are IV and IP injection (Table 1). IV administration has the advantage of delivering therapeutics directly to the bloodstream with the potential for interaction with immune cells and systemic delivery to the entire body, but retention in the bloodstream is not long (especially for whole cells which get trapped in the lung). IP administration has the advantage of allowing tolerance of a larger dose of cells but could be difficult in the presence of abdominal injury. For practical use in military combat situations or for victims of a mass casualty involving radiation injury, the method of administration should be one that requires minimal expertise and equipment in order to benefit a large number of patients in potentially austere conditions.

## 5. Future Directions and Challenges

Despite the promise of cellular therapeutics for the treatment of acute radiation injury, many challenges remain before these therapies can be utilized routinely or stockpiled for emergency use. Future investigation will be necessary to determine how MSCs or other therapies can provide benefits for the treatment of radiation injury, including the level of injury (mild or severe) that might derive the greatest benefit. Careful side-by-side comparisons of live cells and the cell products derived from them (EVs) will be needed to determine potency and efficacy. Since a cell-free product will likely be simpler for use in military treatment facilities or for stockpiling for a mass casualty event, much of the effort could be focused on these products provided they show reasonable efficacy compared with live cell products. Additional work is needed to determine the optimal timing, dosing strategy, and administration methods for both cells and cell-free products. It will be imperative to determine the safety and efficacy of therapeutic agents in patients with different types of combined injury, as well as whether the use of these agents will interfere with the accepted clinical interventions for wound or burn trauma. Another important area for study concerns combination therapies; cellular therapies may be best used in conjunction with other agents, such as antibiotics or growth factors, to improve overall outcomes. More investigation into the mechanisms of action is also warranted. If specific properties of cells or specific combinations of cell-produced factors are identified as necessary and/or sufficient for activity, then cell-free treatments containing the important factors can be developed as a more consistent product, standardized for use in a mass casualty event or possibly tailored to patient-specific needs in less urgent conditions.

## 6. Conclusions

Cellular therapies to prevent, mitigate, or treat acute radiation injury could provide tremendous benefits based on the ability of MSCs or other progenitor cells to reduce inflammation, positively modulate immune function, enhance angiogenesis, and promote recipient regenerative processes. Live cells are very complex biological products that have yet to be optimized for growth, production, storage, administration, or patient selection. For military use or for stockpiling against future mass casualty events, cell-free products such as the secretome or EVs are needed, but the choice of cells, handling of cells during secretome/EV collection, processing methods, storage conditions, and administration methods also require optimization. It still remains to be seen whether secretome or EVs function similarly to live cells for the treatment of acute radiation injury, and this important question should be a priority for future research and development. The use of cells or the cell-free products they produce will likely provide a powerful tool for mitigation and treatment of serious radiation injury, saving lives and reducing long-term damage in those exposed to high radiation doses.

## Figures and Tables

**Table 1:** Common animal species used for investigation of radiation injury.

Species	Advantages	Disadvantages
Mouse	Well-characterized	Physiology distinct from human
	Multiple inbred strains	No prodromal phase
	Genetic modification feasible	Skin wound healing different from human
	Many reagents available	
	Short generation time	
	Lower costs	
Rat	Low costs similar to mouse	Fewer reagents available than for mouse
	Larger size better for surgical studies	More difficult to genetically modify
	More blood and tissue available	Less genetically homogenous than mouse
Guinea Pig	Rapid response to radiation	Less characterized
		Fewer reagents available than for mouse or rat
Ferret	Good model for prodromal phase	Less characterized
		Fewer reagents available than for mouse or rat
Rabbit	Easy to handle	Less characterized
		Fewer reagents available than for mouse or rat
Swine	Good model for skin wounds, burns	More expensive to purchase and maintain
	Good model for H-ARS	More difficult to use large number of animals
		Fewer reagents available than for mouse or rat
Canine	Medium size	Pulmonary system different from human
	Long lifespan	Companion animal status
	Good model for GI-ARS	
	Immune system similarities to human	
NHP	Physiology most comparable to human	More sentient species
	Well-characterized	More expensive to purchase and maintain
	Radiation response similar to human	More difficult to use large number of animals
	Gold standard model for FDA	

Sources: Singh et al. [17], Hunter et al. [18], Williams et al. [19], Singh et al. [20].

**Table 2:** Rodent studies utilizing cellular therapies for the treatment of radiation injury. IV, intravenous; IP, intraperitoneal; RO, retro-orbital; MSC, mesenchymal stromal cell; BM, bone marrow; EV, extracellular vesicle; EC, endothelial cell; ASC, adipose stromal cell; UCB, umbilical cord blood; DPSC, dental pulp stromal cell; CM, conditioned medium.

Reference	Animal Model	Cellular Therapeutic	Admin. Method	Timing	Results/Outcomes
Lange, 2011 [121]	mouse	mouse BM-MSCs	IV	within 8 h	Increased survival, accelerated hematopoeitic recovery
	whole body rad			of irrad	
Yang, 2012 [122]	mouse	mouse MSCs	IV	16–24 h	Increased survival
	whole body rad			post-irrad	
Francois, 2012 [123]	mouse	mouse MSCs	IP	1 day	Increased survival, intestinal epith damage reduced;
	whole body rad	(non-MHC matched)		post-irrad	antiobiotics improved survival
Schoefinius, 2017 [126]	mouse	mouse BM-MSCs/MSC-EVs	IV	shortly after	MSCs increased short & long-term survival;
	whole body rad			post-irrad	EVs only increased long-term survival
Piryani, 2019 [125]	mouse	mouse ECs/EC-EVs	IV	4 daily doses	Increased survival, increased BM cellularity
	whole body rad			post-irrad	
Chinnapaka, 2021 [124]	mouse	mouse ASCs	IP	24 h	Increased survival, accelerated hematopoeitic recovery;
	whole body rad			post-irrad	cells migrated to BM
Chinnadurai, 2021 [81]	mouse	mouse MSCs (activated)	IP	1 day & 8 days	Increased survival
	whole body rad			post-irrad	
Kovelenko, 2013 [127]	mouse	human UCB-MNCs	RO	4 doses @24–52 h	Increased survival
	whole body rad			post-irrad	
Shim, 2013 [128]	mouse	human UCB-MSCs	IV	4 h	Accelerated hematopoietic & BM recovery
	whole body rad			post-irrad	
Wen, 2016 [129]	mouse	human MSC-EVs	IV	6–72 h	Increased WBC & granulocyte numbers at 3 weeks
	whole body rad			post-irrad	
Bouchlaka, 2017 [130]	mouse	human macrophages	IV	3 h	Increased/prolonged survival
	whole body rad	(after MSC co-culture)		post-irrad	
Kim, 2018 [131]	mouse	human UCB-MSCs	IV	3 h or 30 h	Increased survival, accelerated hematopoeitic recovery;
	whole body rad			post-irrad	Increased prolif in BM
Kink, 2019 [132]	mouse	human macrophages	IV	4 h	Increased survival, hematopoeitic recovery if macrophages
	whole body rad	(MSC-EV-educated)		post-irrad	pretreated with EVs from LPS-primed human BM-MSCs
Diaz, 2020 [64]	mouse	human BM-MSCs	RO	3 h or 30 h	Increased survival, improved gut recovery,
	whole body rad			post-irrad	no effect on pancytopenia
Forsberg, 2021 [133]	mouse	human monocytes	IV	4 h, 24 h, 48 h	Increased survival, hematopoetic recovery if injected at 4–24 h
	whole body rad	(MSC-EV-educated)		post-irrad	IL-6 required for female mice but not male mice
Kong, 2021 [134]	mouse	human DPSC-EVs	IV	7 daily doses	Inhibited decrease in WBCs, better recovery at 19–25 days
	whole body rad			post-irrad	
Chen H, 2015 [135]	rat	rat MSC-CM ± activation	IP/IV	IP Continuous	Improved intestinal damage & survival;
	partial rad (abd)			& IV for 3 days	activated MSC-CM more potent
Chen Y-X, 2015 [136]	rat	rat MSC-CM	IV	Pre-irradiation	Improved liver pathology
	partial rad (liver)				
